# The Safety of Immunosuppressants Used in the Treatment of Immune-Related Adverse Events due to Immune Checkpoint Inhibitors: a Systematic Review

**DOI:** 10.7150/jca.87335

**Published:** 2023-09-11

**Authors:** Antonio Pizuorno Machado, Hunter Ratliff, Ahmed Abdelwahab, Muhammad H. Vohra, Andrew Kuang, Malek Shatila, Muhammad Ali Khan, Menhaz A. Shafi, Anusha S. Thomas, Jessica Philpott, Omar Alhalabi, Yinghong Wang

**Affiliations:** 1 Department of Internal Medicine, The University of Texas Health Science Center, Houston, TX, USA.; 2 Department of Gastroenterology, Hepatology, and Nutrition, The University of Texas MD Anderson Cancer Center, Houston, TX, USA.; 3 Department of Medicine, Baylor College of Medicine, Houston, TX, USA.; 4 Inflammatory Bowel Disease Center, Cleveland Clinic, Cleveland, OH, USA.; 5 Department of Genitourinary Medical Oncology, The University of Texas MD Anderson Cancer Center, Houston, TX, USA.

**Keywords:** immune checkpoint inhibitor, immune-related adverse events, immunosuppressant, complications, infection, meta-analysis

## Abstract

**Purpose:** Immune checkpoint inhibitor (ICI) use can lead to immune-related adverse events (irAEs) that require treatment with immunosuppressive medications in moderate to severe cases. Oncology society guidelines recommend systemic steroids and immunosuppressants such as infliximab and vedolizumab for the treatment of refractory cases. Limited information is available about the safety profile and potential adverse effects of these immunosuppressants. We have investigated the safety profile of multiple immunosuppressants which are used in the treatment of ICI-related irAEs.

**Methods:** We performed a systematic review of studies reporting irAEs, from ICI use, and their medical management with immunosuppressants in adult cancer patients. We searched MEDLINE, EMBASE, Cochrane Library, and ClinicalTrials.gov from inception through September 1, 2022, using the following keywords or their equivalents: ICI, immunosuppressant, and irAE. We extracted observational studies and clinical trials that matched our criteria. A random effects model was used to estimate the overall incidence of infections associated with the treatment of irAEs.

**Results:** Among the 11 studies included in this review (1036 total patients), melanoma (548 patients, 52.9%) was the most common primary cancer, followed by lung cancer (139 patients, 13.4%) and genitourinary cancers (131 patients, 12.6%). PD-1/PD-L1 monotherapy (460 patients, 44.4%) was used most, followed by a combination of PD-1/PD-L1 and CTLA-4 therapy (350 patients, 33.8%) and CTLA-4 monotherapy (226 patients, 22%). A total of 1024 (98.8%) patients had their irAEs treated with systemic steroids with majority having colitis and hepatobiliary irAEs; 335 patients (32.3%) were also treated with infliximab (mainly for colitis). Our review found 22.3% of patients treated for irAEs developed infectious adverse events (95% CI: 15.6%-29.1%, p<0.001). Among the 3 studies reporting the types of infections (41 total patients), bacterial (80.5%), followed by fungal (36.6%), infections were most common.

**Conclusions:** Adverse events from irAE treatment occurred in about one-third of patients that received either steroids or a combination of steroids and other immunosuppressants. Clinicians should be aware of these immunosuppressant-related adverse effects, which can negatively impact cancer treatment and patient outcomes, when treating irAEs and consider shortening treatment duration or using alternative strategies when possible to mitigate these complications, future prospective studies should further investigate the safety of immunosuppressants in treating irAEs.

## Introduction

Immune checkpoint inhibitors (ICIs) have become the standard of care in the management of several cancers 1. With increased utilization of ICIs, clinicians have noted an increased incidence of immune-related adverse events (irAEs) during cancer treatment 2. These irAEs affect multiple organs, including the gastrointestinal tract, lungs, thyroid, and skin. The involvement of the heart and the nervous system is rare but is associated with a higher mortality. 3. These adverse effects are the main barrier to using ICIs in clinical care 4. For example, in a large retrospective study, 61.8% of patients who received anti-programmed cell death protein 1/programmed death ligand 1 (PD-1/PD-L1) experienced irAEs, resulting in 46.6% of the study population to discontinue treatment 5. Further, a large meta-analysis of 112 clinical trials reported fatality rates of these toxicities to be 0.36% for patients treated with anti PD-1 therapy and up to 0.38% for patients treated with anti PD-L1 therapy 6. Compared with patients who received PD-1/PD-L1 inhibitors, patients who received the cytotoxic T-cell lymphocyte-associated antigen 4 (CTLA-4) inhibitor ipilimumab (as monotherapy or in combination with other ICIs) had a higher incidence of irAEs 7. Early detection and effective management of these adverse effects can help clinicians and patients consider ICI re-challenge and improve overall patient outcomes. Notably, certain irAEs such as colitis are more common than the other organs, such as lung, heart or endocrine 8. The clinical practice also differs among many academic or practice groups, which could lead to wide variety of medical treatment for irAEs 9.

Systemic immunosuppression is frequently used to treat moderate to severe irAEs, but it increases the potential risk for opportunistic infections and may negatively affect anti-tumor immunity 10-11. Patients with grade ≥ 2 irAEs or multiple concurrent irAEs often require steroids treatment 12. Multiple oncology societies have published guidelines on the appropriate evaluation and management of irAEs 13-17. These guidelines note that steroids and other immunosuppressants, including tumor necrosis factor-alpha inhibitors (such as infliximab), play a key role in managing irAEs for patients with steroid-refractory and steroid-dependent irAEs. Accordingly, the early introduction of infliximab or vedolizumab during disease course in patients with colitis has shown favorable clinical outcomes 18.

However, these medical treatments also have side effects of their own. The 2022 National Comprehensive Cancer Network guidelines cautioned against using infliximab in patients with reduced left ventricular ejection fraction, with the concern of potential precipitation of heart failure 15, 19. Less commonly, infliximab was also associated with other potential toxic effect such as hepatotoxicity, and skin rash etc 13, 20, 21. As a commonly recognized side effects, immunosuppressant use was also attributable to increased risks of hepatitis B infection, tuberculosis reactivation, and infections leading to hospitalization and possibly death 15, 21-23. Moreover, it has been in the discussion that steroid and biologic agents may negatively impact the cancer patients' survival, although multiple confounding factors need to be taken into consideration when interpreting this data especially given the lack of high-quality prospective randomized data in this field 22, 24. On the other hand, the use of biologic agents/steroid has been standard over the years for patients with inflammatory bowel disease (IBD) with acceptable safety profile despite the prolonged treatment duration (induction and maintenance) compared to frequent short treatment course for immune-related colitis (<3 doses) 25, 26. However, given the different disease nature and context of malignancy, the safety of the immunosuppressant including biologics may differ and certainly warrant further investigation. In this systematic review, we investigated the safety of common immunosuppressive medications, including systemic corticosteroids and other immunosuppressants (infliximab, vedolizumab, tocilizumab, tacrolimus, alemtuzumab, leflunomide, ustekinumab, and rituximab), used to treat ICI-related irAEs with a deeper dive on the literature summary and analysis to guide clinical management which has been lacking previously.

## Methods

### Data sources and study selection

In accordance with the Preferred Reporting Items for Systematic Reviews and Meta-Analyses (PRISMA) 27 reporting guideline, two authors (APM and AA) independently searched the bibliographic databases MEDLINE, PMC, EMBASE, and Cochrane Library and the trial registry ClinicalTrials.gov from inception through September 1, 2022. All databases were searched for publications containing terms related to ICI agents and irAEs in their title and/or abstract (see Supplemental Methods and tables). Only studies with available full-text articles that were published in peer-reviewed journals were screened for the final analysis.

Observational (retrospective and prospective) studies and interventional trials fulfilling the following PICO criteria (Participants, Intervention, Comparison, and Outcome) were included. Participants were adult patients with any cancer who had been treated with PD-1/PD-L1 and/or CTLA-4 inhibitors. The intervention and exposure criteria were that patients were treated with an ICI and diagnosed with an irAE(s) for which they received immunosuppressants. No comparisons were made. Outcomes were complications from use of immunosuppressants.

To avoid counting the same patients multiple times, if multiple studies were suspected to share the same patient population (e.g., they were conducted at the same site with similar inclusion criteria), only the most recently published study was included. Five independent reviewers (APM, MS, AA, MHV, AK) examined all selected studies, and a mutual consensus was reached.

### Data extraction and synthesis of results

Five researchers (APM, MS, AA, MHV, AK) independently extracted data from the included studies into Excel 2020 (Microsoft) using a standardized form. The types of data extracted are listed in the Supplemental Methods and tables.

Data extraction was reviewed by two independent individuals (APM, HR). Any discrepancies in individual conclusions were resolved by a joint reassessment to reach a consensus. Although we did not conduct a formal meta-analysis (due to the clinical and methodologic heterogeneity of studies identified in this review), we have summarized the studies' general characteristics and shared outcomes for descriptive purposes. In this exploratory analysis, we used random effects models to estimate the overall rates of infections among the studies reporting this information, this was done with SPSS v. 26.

## Results

### Study selection

**Figure 1:**
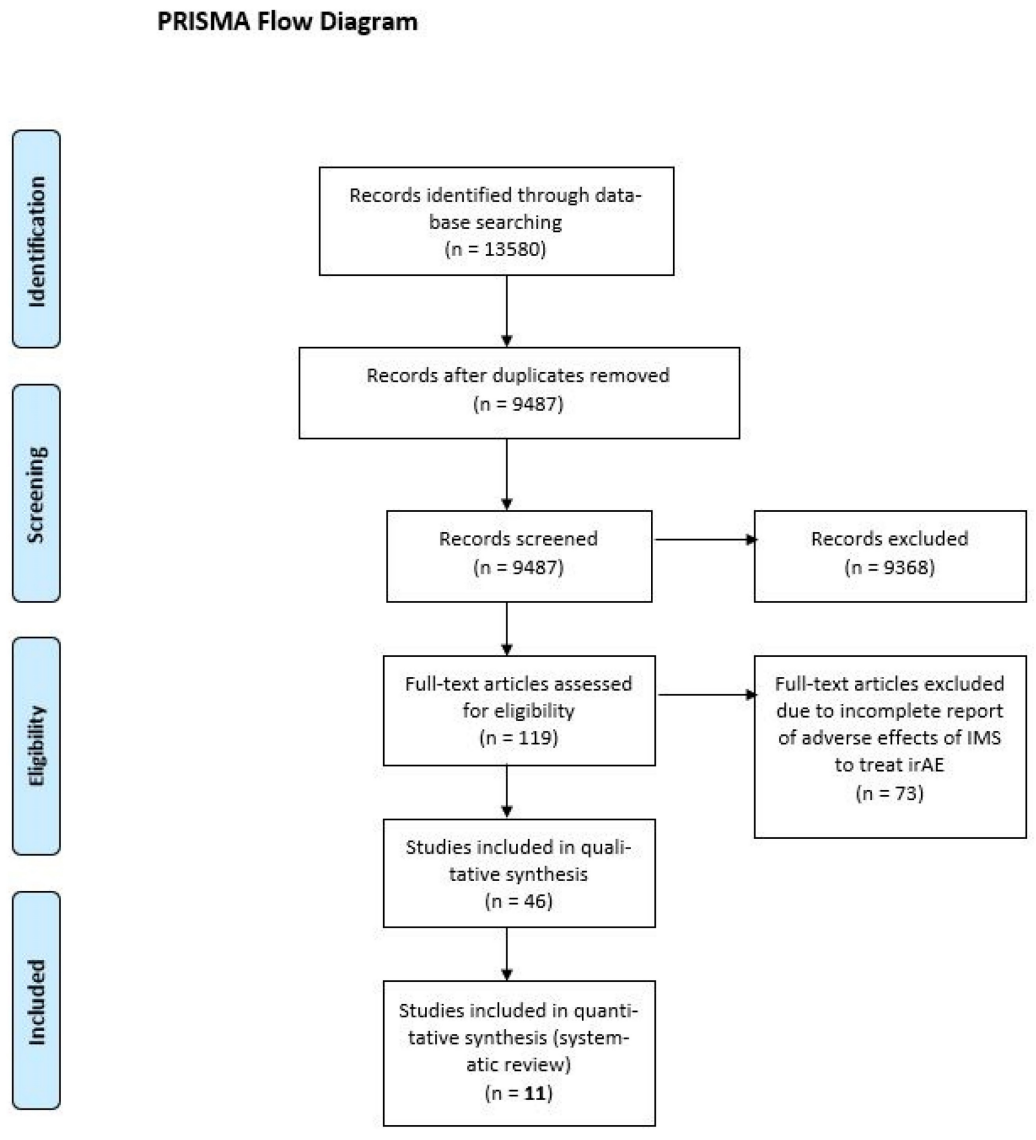
PRISMA flow diagram. IMS, immunosuppressant; irAE, immune-related adverse event.

Our database search yielded 13,580 results; after duplications were excluded, 9487 articles were considered. Of the considered articles, 119 articles were identified for full-text review, of these 73 articles did not meet our inclusion criteria. Of the 46 remaining articles, 11 studies were included in the final analysis, as the others did not assess the safety of irAE treatments (Figure 1) 22, 23, 28-35.

### Study characteristics

All included articles were retrospective studies of patients who experienced irAEs related to ICI use. Most of these investigations focused on specific types of irAEs; gastrointestinal irAEs 22, 23, 30, 35, followed by hepatic irAEs 28, 32, were most common (Table 1). However, 3 studies included patients experiencing all irAE 31, 32, 36. Treatment with systemic corticosteroids was a prerequisite for inclusion in many studies 22, 23, 29-30, 32, 33, 36. Two studies examined steroid-refractory and/or steroid-resistant irAEs and required patients to be treated with corticosteroids and additional immunosuppressants.

### Patient characteristics

Among the 1036 patients examined across all 11 studies, the most common primary cancer was melanoma, which was present in 52.9% of patients, followed by lung (13.4%) and genitourinary (12.6%) cancers (Table 2). Patients received a wide array of ICI treatments; PD-1/PD-L1 inhibitor monotherapy (460 patients, 44.4%) was most common, followed a combination of PD-1/PD-L1 inhibitors and CTLA-4 (350 patients, 33.8%) and CTLA-4 monotherapy (226 patients, 21.8%).

Most irAEs involved the gastrointestinal tract and hepatobiliary tract (Table 3). Colitis was present in 448 patients (43.2%), followed by hepatic cholangiopathy in 398 patients (38.4%), dermatological irAEs such as rash in 146 patients (14.1%), and pneumonitis in 72 patients (6.9%).

The majority of patients in our review received steroids as treatment for irAEs irrespective of the type or severity (98.8%). Steroids were most frequently administered with infliximab, than alone or in combination with another immunosuppressant 22, 23, 29-31, 35. Infliximab was used in 335 patients (32.3%); this was followed by vedolizumab 23 in 77 patients (7.4%) and mycophenolate in 53 patients (5.1%). Mycophenolate was also used to treat pneumonitis in conjunction with infliximab 29. Methotrexate and adalimumab were used infrequently.

### Adverse events from treating irAEs

**Figure 2:**
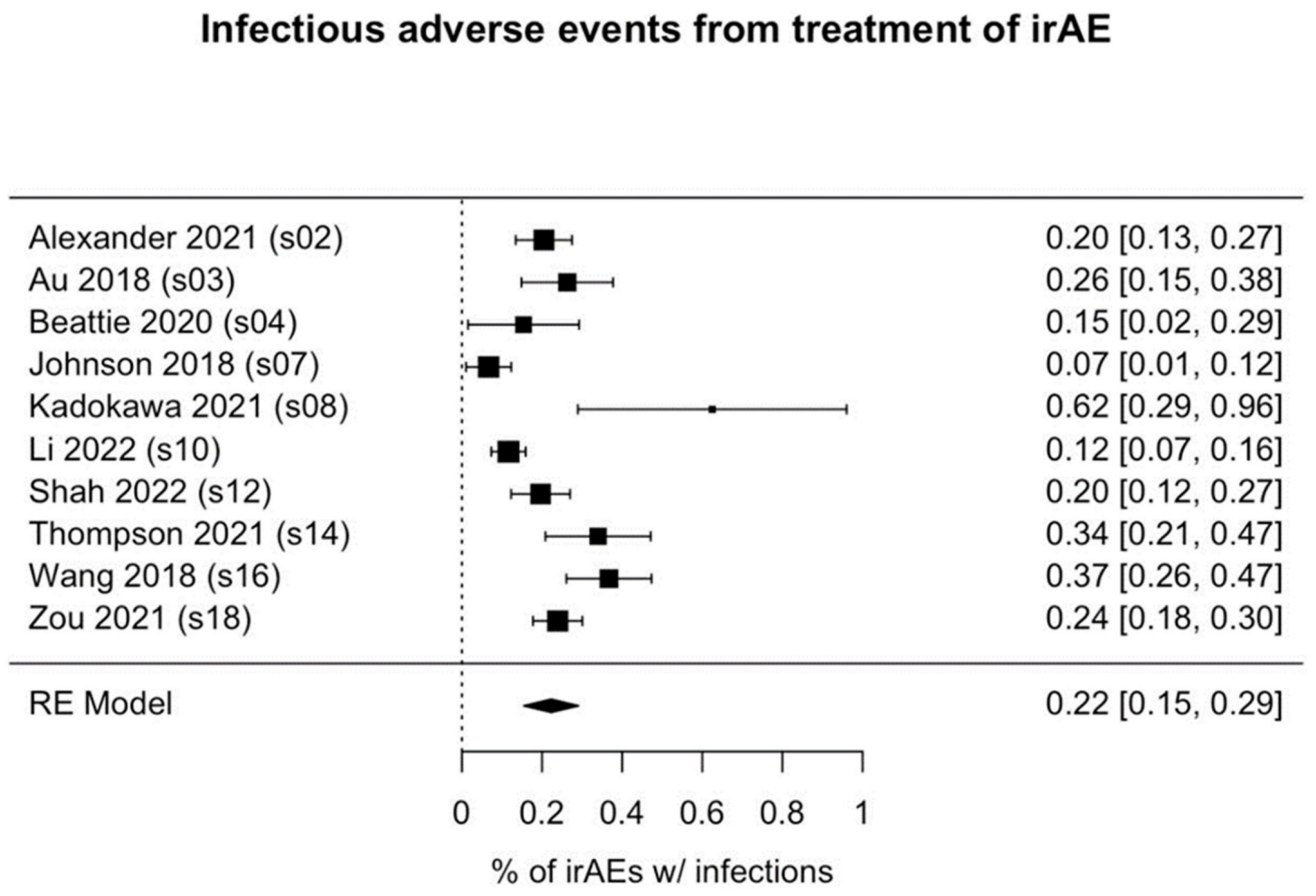
Forest plot of pooled rate of infectious adverse events from immunosuppressant treatment of irAE(s). Each box's placement along the axis indicates the proportions of patients in the study who experienced infections; the horizontal lines represent 95% confidence intervals. The size of each box indicates its weight in the analysis. Results are shown for 10 of the 11 studies we analyzed; in 1 study (Williams et al.), no infections were reported. irAE, immune-related adverse event.

Of the 1036 patients who received immunosuppressant treatment for irAEs, 231 (22.3%) had infectious adverse events (95% CI: 15.6%-29.1%, p<0.001; Figure 2). Among the 3 studies reporting the types of infections observed (41 patients), bacterial infections (occurring in 80.5% of patients) were most common, followed by fungal infections (in 36.6% of patients). Notably, the rates of infection were highly variable among the studies, ranging from as high as 50% 31 to as low as 2.7% 30.

## Discussion

ICIs have been widely employed over the last decade due to their highly efficacious anti-tumor activity. However, their association with irAEs has led to significant morbidity and mortality. In this systematic review, we investigated the safety of common immunosuppressants used to treat ICI-related irAEs. Our analysis showed that patients who received immunosuppressants to treat gastrointestinal and hepatobiliary irAEs experienced additional adverse events, namely, an overall infection rate of 22.3%, that emphasize the need for thorough scrutiny and early recognition of these complications.

Given the wide array of irAEs in different body systems, it is possible that those irAEs could predispose patients to secondary infections regardless of immunosuppressant treatment. This hypothesis has been proposed and evaluated in several investigations. Previous evidence suggests that ICI-related colitis increases susceptibility to gastrointestinal tract infections such as *Clostridioides difficile* 37, non-*C. diff.* bacterial, and viral infections 38. Infections, including Cytomegalovirus infections, have also been reported in the extra-gastrointestinal tract after ICI exposure, some of which could bear fatal outcomes such as pneumonitis 39. However, it is still unclear if the underlying inflammatory adverse event predisposes patients to a higher risk of infection and colonization or if the pathogen itself contributes to a worse inflammatory process, leading to impaired outcomes. Regardless, it is imperative for clinicians to identify the infection early during irAE evaluation and management to ensure prompt therapy 40.

Certain immunosuppressant treatments can predispose patients to a reactivation of varicella-zoster infection, latent tuberculosis, or hepatitis infections. Our analysis did not encounter any patients with these complications, which might be explained by the annual and/or frequent screening of cancer patients for these types of infections 41.

Whether immunosuppressant treatment has an impact on cancer outcome and survival remains unclear due to conflicting evidence. A recent meta-analysis 42 reported that exposure to corticosteroids for any indication prior to ICI therapy did not jeopardize survival outcomes or treatment response 43. However, Burdett et al. showed that patients receiving steroids to treat irAEs had heterogeneous results regarding their cancer outcomes 44. Different groups have reported that patients with irAEs treated with systemic immunosuppressants and patients without irAEs have similar cancer outcomes 45-47. Yet, there is also evidence to suggest that treatment with a steroid plus anti-tumor necrosis factor for steroid-refractory irAEs led to a significantly lower survival rate than steroid use alone did 24. Unfortunately, high-quality data in this area are scarce; further study is needed.

A key limitation of our systematic review is the heterogeneity of the identified studies. The studies varied greatly in their aims and objectives, inclusion criteria, ICI exposure, and safety reporting. We were unable to review cancer-related outcomes, including cancer progression, progression-free survival, overall survival, and mortality, in detail due to inconsistencies in reported key safety outcomes related to immunosuppressant use. Secondly, most studies did not report key factors that may influence a patient's predisposition to infections (e.g., use of prophylactic antimicrobials, other comorbidities, recent surgeries, time of presentation, characteristics of the host, steroid dosage and treatment duration) and other confounding factors, which may account for the high variability of infectious complications observed in these studies. Thirdly, given the low incidence of certain irAEs and the less requirement for immunosuppressant treatment, the majority of the articles we extracted was focusing on GI and hepatobiliary AEs, which potentially put limitation on the generalizability of its findings. These are common limitations of systematic reviews and meta-analyses. It is imperative to establish a systematic approach for clinicians to routinely report adverse events from management of irAEs given the rapidly emerging use of the immunosuppressive medications.

In conclusion, complications from therapy for irAEs occurred in about less than one-third of patients that required treatment with either steroids or a combination of steroids and other immunosuppressants. Clinicians should therefore be aware of this increased risk and consider minimizing immunosuppressant treatment or using alternative therapy when available to avoid interruptions or negative impact in cancer care. Future prospective studies are needed to further investigate the safety of immunosuppressants in treating irAEs.

## Supplementary Material

Supplementary information.

## Figures and Tables

**Table 1:** Characteristics of studies included in the analysis.

Study information		Inclusion criteria		Treatment for irAE
First author and year	Study design	ICI-related irAE	Minimum treatment	Treatment arms
Alexander 2021	RCS	Colitis	CS-refractory; IFX	CS + IFX
Au 2018	RCS	Hepatitis	Any	Variety*
Beattie 2020	RCS	Pneumonitis	CS-refractory; IMS	CS + IFX +/- Mycophenolate
Johnson 2018	RCS	Colitis	CS	CS, CS + IFX
Kadokawa 2021	Case series	Any	Any	CS + IFX
Li 2022	RCS	Hepatitis	CS	CS
Shah 2022	RCS	Any	CS	CS + Variety*
Thompson 2021**^‡^**	RCS	Rash	Any	CS
Wang 2018	RCS	Colitis	Any	CS, CS + IFX
Williams 2019	RCS	Any	CS	CS
Zou 2021	RCS	Colitis	CS	CS, CS + IFX, CS + VDZ, CS + IFX + VDZ

CS, corticosteroid; ICI, immune checkpoint inhibitor; IFX, infliximab; IMS, immunosuppressant; irAE, immune-related adverse event; RCS, retrospective cohort study; TNF, tumor necrosis factor; VDZ, vedolizumab. *Includes anti-TNF, mycophenolate, and anti-thymocyte globulin. ‡Our analysis for this study was limited to those who received systemic corticosteroids. *Includes anti-TNF, mycophenolate, and anti-thymocyte globulin. ‡Our analysis for this study was limited to those who received systemic corticosteroids.

**Table 2:** Cancer characteristics of and type of ICI received by patients included in the analysis.

Study information		Primary cancer			Type of ICI used		
First author and year	Patientsn	Melanoma n (%)	Lungn (%)	GUn (%)	PD-1/PD-L1 n (%)	CTLA-4 n (%)	Combinationn (%)
Alexander 2021	127	90 (70.8)	7 (5.5)	9 (7.1)	40 (31.5)	21 (16.5)	66 (52.0)
Au 2018	57	NR	NR	NR	21 (36.8)	0 (0)	36 (63.2)
Beattie 2020	26	4 (15.4)	9 (34.6)	6 (23.1)	19 (73.1)	1 (3.8)	6 (23.1)
Johnson 2018	75	53 (70.7)	2 (2.7)	15 (20)	17 (22.7)	46 (61.3)	12 (16)
Kadokawa 2021	8	4 (50)	3 (37.5)	1 (12.5)	7 (87.5)	0 (0)	1 (12.5)
Li 2022	215	117 (54.4)	23 (10.7)	24 (11.2)	94 (43.7)	27 (12.6)	94 (43.7)
Shah 2022	112	68 (60.7)	22 (19.6)	10 (8.9)	50 (44.6)	25 (22.3)	37 (33.0)
Thompson 2021**^‡^**	50	13 (26)	13 (26)	0 (0)	42 (84)	2 (4)	6 (12)
Wang 2018	79	79 (100)	0 (0)	0 (0)	19 (24.1)	48 (60.8)	12 (15.2)
Williams 2019	103	57 (55.3)	34 (33.0)	5 (4.9)	59 (57.3)	28 (27.2)	16 (15.5)
Zou 2021	184	63 (34.2)	26 (14.1)	61 (33.2)	92 (50)	28 (15.2)	64 (34.8)

CTLA-4, cytotoxic T-cell lymphocyte-associated antigen 4; ICI, immune checkpoint inhibitor; GU, genitourinary; NR, not reported; PD-1/PD-L1, programmed cell death protein 1/programmed death ligand 1. ‡Our analysis for this study was limited to those who received systemic corticosteroids.

**Table 3:** Patient irAE characteristics and incidence rates of infection for patients included in the analysis.

Study information			Type of irAE			Treatment for irAE			Follow up	
First author and year	Patientsn	GIn (%)	Hepatobiliaryn (%)	Lungn (%)	Steroids n (%)	Median steroids dose (mg)	IFX n (%)	VDZn (%)	Infection rate n (%)	Duration (mons)
Alexander 2021	127	127 (100)	0 (0)	0 (0)	127 (100)	> 5 mg	127 (100)	127 (100)	26 (20.5)	6
Au 2018	57	0 (0)	57 (100)	0 (0)	45 (78.9)	1.3 mg/kg	0 (0)	0 (0)	15 (26.3)	4
Beattie 2020	26	0 (0)	0 (0)	26 (100)	26 (100)	100 mg	20 (76.9)	0 (0)	4 (15.4)	2
Johnson 2018	75	75 (100)	0 (0)	0 (0)	75 (100)	NR	36 (48)	0 (0)	2 (2.7)	26
Kadokawa 2021	8	7 (87.5)	1 (12.5)	0 (0)	8 (100)	NR	8 (100)	0 (0)	4 (50)	3
Li 2022	215	55 (25.6)	215 (100)	58 (27.0)	215 (100)	NR	0 (0)	0 (0)	25 (11.6)	NR
Shah 2022	112	48 (42.9)	28 (25)	23 (20.5)	112 (100)	>20 mg	15 (13.4)	3 (2.7)	22 (19.6)	3
Thompson 2021**^‡^**	50	0 (0)	0 (0)	0 (0)	50 (100)	NR	0 (0)	0 (0)	17 (34)	3
Wang 2018	79	79 (100)	0 (0)	0 (0)	79 (100)	NR	35 (44.3)	0 (0)	29 (36.7)	1
Williams 2019	103	0 (0)	0 (0)	28 (27.2)	103 (100)	1mg/kg	0 (0)	0 (0)	NR	3
Zou 2021	184	184 (100)	0 (0)	0 (0)	184 (100)	NR	94 (51.1)	63 (34.2)	35 (19.0)	45

GI, gastrointestinal; IFX, infliximab; irAE, immune-related adverse event; NR, not reported; VDZ, vedolizumab. ‡Our analysis for this study was limited to those who received systemic corticosteroids.
