# Supplementary material for: The Safety of Immunosuppressants Used in the Treatment of Immune-Related Adverse Events due to Immune Checkpoint Inhibitors: a Systematic Review
Source: J Cancer. 2023 Sep 11;14(16):2956–63. doi: 10.7150/jca.87335 (PMC10583582; doi:10.7150/jca.87335)
Supplement: Supplementary file 1 — Supplementary information. [file jcav14p2956s1.pdf]

SUPPLEMENTAL METHODS

Search strategy

The following search queries were used to search the selected databases and trial registries:

1. ICI agents: ("checkpoint inhibitor*" or "checkpoint modulator*" or "checkpoint antibod*" or "checkpoint block*" or ICI* or "CTLA-4" or PD1 or "PD-1" or PDL1 or "PD-L1" or Yervoy or "MDX 010" or MDX010 or "BMS-734016" or BMS734016 or pembrolizumab or Keytruda or Lambrolizumab or "Merck 3475" or "MK 3475" or MK3475 or "Sch 900475" or "HSDB 8257" or nivolumab or "BMS 936558" or BMS936558 or "MDX 1106" or MDX1106 or "ONO 4538" or ONO4538 or Opdivo or MEDI0680 or atezolizumab or Tecentriq or MPDL3280A or "MPDL 3280A" or "RG 7446" or RG7446 or durvalumab or imfinzi or "MEDI 4736" or MEDI4736 or avelumab or Bavencio or MSB0010718C or "MSB 0010718C" or MSB0010682 or "BMS 936559" or BMS936559 or Cemiplimab or libtayo or REGN2810).ti,ab. or ("interleukin-17 blockade" or "ipilimumab-related").ti.

2. irAEs: (Drug Toxicity or Abnormalities, Drug Induced or Drug Hypersensitivity or (ae or to or co).fs. or (safe or safety or "side effect*" or "adverse event*" or "adverse effect*" or irAE* or toxicity or complication* or noxious or tolerability or "side effect*" or ADRs or "ADEs drug surveillance" or harm* or inflam*).ti,ab. or (adverse or undesirable or harms* or serious or toxic) adj2 (effect* or reaction* or event* or outcome*)).ti,ab.

Data extraction

We extracted the following information for each study: aim of the study, study design, total number of patients, mean age of the whole cohort, prevalence of cancer types, use of PD-1/PD-L1 inhibitors and/or CTLA-4 inhibitors (alone or in combination), incidence and

types of irAEs, and use of immunosuppressants (e.g., steroids, infliximab, vedolizumab, tocilizumab). We also recorded the following adverse events of immunosuppressant treatment: incidence of infections, type of infections (i.e., bacterial, viral, fungal), incidence of hepatotoxicity, and incidence of hyperglycemia. We used the Common Terminology Criteria for Adverse Events (CTCAE) version 4.0 to define irAEs from systemic therapy, as most protocols in our review were written before November 2017 (when CTCAE version 5.0 was introduced). According to CTCAE 4.0, the severity of an adverse event is represented by grades 1 through 5, with unique clinical descriptions for each adverse event based on its severity.
